# A combination intervention strategy to improve linkage to and retention in HIV care following diagnosis in Mozambique: A cluster-randomized study

**DOI:** 10.1371/journal.pmed.1002433

**Published:** 2017-11-14

**Authors:** Batya Elul, Matthew R. Lamb, Maria Lahuerta, Fatima Abacassamo, Laurence Ahoua, Stephanie A. Kujawski, Maria Tomo, Ilesh Jani

**Affiliations:** 1 ICAP at Columbia University, Mailman School of Public Health, Columbia University, New York, New York, United States of America; 2 Department of Epidemiology, Mailman School of Public Health, Columbia University, New York, New York, United States of America; 3 Center for Collaboration in Health, Maputo, Mozambique; 4 Instituto Nacional de Saúde, Maputo, Mozambique; University of Melbourne, AUSTRALIA

## Abstract

**Background:**

Concerning gaps in the HIV care continuum compromise individual and population health. We evaluated a combination intervention strategy (CIS) targeting prevalent barriers to timely linkage and sustained retention in HIV care in Mozambique.

**Methods and findings:**

In this cluster-randomized trial, 10 primary health facilities in the city of Maputo and Inhambane Province were randomly assigned to provide the CIS or the standard of care (SOC). The CIS included point-of-care CD4 testing at the time of diagnosis, accelerated ART initiation, and short message service (SMS) health messages and appointment reminders. A pre–post intervention 2-sample design was nested within the CIS arm to assess the effectiveness of CIS+, an enhanced version of the CIS that additionally included conditional non-cash financial incentives for linkage and retention. The primary outcome was a combined outcome of linkage to care within 1 month and retention at 12 months after diagnosis. From April 22, 2013, to June 30, 2015, we enrolled 2,004 out of 5,327 adults ≥18 years of age diagnosed with HIV in the voluntary counseling and testing clinics of participating health facilities: 744 (37%) in the CIS group, 493 (25%) in the CIS+ group, and 767 (38%) in the SOC group. Fifty-seven percent of the CIS group achieved the primary outcome versus 35% in the SOC group (relative risk [RR]_CIS vs SOC_ = 1.58, 95% CI 1.05–2.39). Eighty-nine percent of the CIS group linked to care on the day of diagnosis versus 16% of the SOC group (RR_CIS vs SOC_ = 9.13, 95% CI 1.65–50.40). There was no significant benefit of adding financial incentives to the CIS in terms of the combined outcome (55% of the CIS+ group achieved the primary outcome, RR_CIS+ vs CIS_ = 0.96, 95% CI 0.81–1.16). Key limitations include the use of existing medical records to assess outcomes, the inability to isolate the effect of each component of the CIS, non-concurrent enrollment of the CIS+ group, and exclusion of many patients newly diagnosed with HIV.

**Conclusions:**

The CIS showed promise for making much needed gains in the HIV care continuum in our study, particularly in the critical first step of timely linkage to care following diagnosis.

**Trial registration:**

ClinicalTrials.gov NCT01930084

## Abstract 2

In a cluster-randomized trial done in Mozambique, Batya Elul and colleagues study a combined intervention for linkage to and retention of people with HIV in care.

## Author summary

**Why was this study done?:**

- In sub-Saharan Africa, HIV testing, care, and treatment programs have been widely scaled up over the past decade, but suboptimal outcomes across the HIV care continuum—particularly with regards to timely linkage to and sustained retention in care—compromise their effectiveness.
- Patients experience multiple barriers to linkage to and retention in HIV care including health system barriers, structural barriers, and behavioral barriers, yet prior studies have largely evaluated individual interventions targeting a single barrier to care.
- Our study was designed specifically to examine the effectiveness of a combination intervention strategy (CIS) composed of several scalable evidence-based interventions targeting the multiple and prevalent health system, structural and behavioral barriers that patients face across the HIV continuum.

**What did the researchers do and find?:**

- We randomly assigned 10 primary health facilities in the city of Maputo and Inhambane Province in Mozambique to provide the standard of care (SOC) or the CIS, which included point-of-care CD4 testing at the time of diagnosis, accelerated ART initiation, and short message service (SMS) health messages and appointment reminders. A pre–post intervention 2-sample design was nested within the intervention arm to assess the effectiveness of CIS+, an enhanced version of the CIS that additionally included conditional non-cash financial incentives for linkage and retention.
- We enrolled 2,004 adults diagnosed with HIV in the voluntary counseling and testing clinics of participating health facilities, and compared the proportion who achieved a combined outcome of linkage to HIV care within 1 month of diagnosis and retention in care at 12 months across the 3 study groups.
- We found an increased likelihood of achieving the combined outcome in the CIS group compared to the SOC group, driven primarily by very large increases in same-day linkage, but no difference between the CIS+ and CIS groups.

**What do these findings mean?:**

- The CIS may help improve outcomes across the HIV care continuum in high-burden settings, particularly in the critical first step of timely linkage to care following diagnosis.
- Further research is needed to understand whether financial incentives can be optimized in this setting, given their effectiveness in enhancing other health outcomes.

## Introduction

Although the extraordinary scale-up of HIV testing, care, and treatment programs in sub-Saharan Africa over the past decade has resulted in more than 19 million persons accessing antiretroviral therapy (ART) [1], the effectiveness of these programs has been significantly hindered by high levels of attrition across the HIV care continuum. Observational studies and systematic reviews have repeatedly reported disturbing gaps in care as patients move from HIV testing clinics to HIV care clinics (i.e., linkage to care) and that patient dropout among those enrolled in HIV care is far too common, both before and after ART initiation (i.e., retention in care) [2–7]. Indeed, available data suggest that less than 1/3 of individuals who are diagnosed with HIV are successfully linked to and remain engaged in HIV care 12 months later [4,8].

Barriers to timely linkage to and sustained retention in HIV care have been well documented, and include health system barriers (e.g., multiple HIV clinic visits for counseling and clinical and laboratory assessments prior to ART initiation), structural barriers (e.g., transport costs and distances, work and childcare constraints), and behavioral barriers (e.g., forgetting appointments, lack of understanding of required care) [9–14]. Prior studies have overwhelmingly evaluated individual interventions targeting a single barrier at a single point in the HIV care continuum such as mobile phone short message service (SMS) messaging to augment linkage to care following diagnosis, or point-of-care CD4 testing to enhance retention among patients enrolled in HIV care [15,16]. However, it is increasingly recognized that multi-component approaches composed of several practical, evidence-based interventions that simultaneously target the multiple and recurrent barriers that patients face as they navigate across the HIV care continuum are needed to maximize individual and population health [17,18]. Further, implementation science research that evaluates proposed multi-component approaches in real-world settings is needed to assess not only effectiveness, but also implementation outcomes including reach, adoption, and sustainability [19]. To this end, we designed a combination intervention strategy (CIS) composed of several scalable evidence-based interventions targeting prevalent health system, structural, and behavioral barriers across the HIV care continuum, and determined its effect on a combined outcome of linkage to and retention in HIV care among adults newly diagnosed with HIV in Mozambique, while also collecting information on its implementation and potential for broader scale-up [20]. Data regarding intervention feasibility and patient acceptability have been published [21], and thus we present here the effectiveness results. Because the interventions included in the CIS are expected to be implemented at the facility level, as opposed to targeted at specific individuals, should they be scaled up, we evaluated effectiveness using a cluster design, which best mirrors this implementation approach.

## Methods

A detailed description of the study protocol has been published [22].

### Ethics statement

Ethical approval was provided by Mozambique’s National Committee for Bioethics for Health and Columbia University’s institutional review board (IRB) (protocol AAAL1354). Informed written consent was obtained from all participants.

### Study design

Between April 22, 2013, and June 30, 2016, we conducted a 2-arm cluster-randomized study (effectiveness–implementation hybrid design, Type 1) [20] in health facilities in Maputo and Inhambane Province in Mozambique in order to assess the effectiveness of the CIS. Additionally, a pre–post intervention 2-sample design was nested within the intervention arm to assess the additional effectiveness of an enhanced version of the CIS, referred to as CIS+. Consequently, the standard of care (SOC) arm enrolled 1 cohort of patients, while the intervention arm enrolled 2 sequential cohorts of patients (CIS and CIS+). CIS+ participants were enrolled after CIS enrollment was completed at each facility randomized to the intervention arm.

### Study setting

The city of Maputo, the nation’s capital, has an area of 300 km^2^ and an estimated population of 1,225,868 [23], with an HIV prevalence of 16.9% among those aged 15 to 59 years [24]. The Maputo City Health Network has a total of 37 health facilities, 32 of which offered comprehensive HIV care and treatment services at the time of study implementation [25]. In contrast, Inhambane is a rural province, with an estimated 1,475,318 people spread across 68,615 km^2^ [23]. HIV prevalence among adults aged 15 to 59 years is 14.1% [24]. The ratio of doctors to population (5.96/100,000) is one of the lowest in the country [26]. Of the 135 health facilities in the province, 76 offered HIV care and treatment services when our study was initiated [25]. Suboptimal health facility infrastructure, long distances to facilities, and weak referral systems in the province are all believed to compromise health service uptake [26].

### Randomization

Primary health facilities providing HIV testing, care, and treatment services and operated by the Ministry of Health with technical support from the Center for Collaboration in Health, a local PEPFAR implementing partner, were the unit of randomization. We focused on primary health facilities, rather than larger provincial hospitals, to reflect the increasingly decentralized nature of HIV service delivery in Mozambique. Ten facilities in Maputo (*N =* 4) and Inhambane Province (*N =* 6) were selected from the 66 primary health facilities receiving technical support from the Center for Collaboration in Health in those regions. Participating facilities were purposely chosen because they had the highest volume of adults testing HIV positive and enrolling in HIV care in the year prior to study start and thus were expected to have sufficient participants for appropriate power. Facilities were matched into pairs by region (Maputo or Inhambane), level of urbanicity (urban versus rural), and average number of patients testing HIV positive in voluntary counseling and testing (VCT) in the year prior to study initiation (high versus low), resulting in 5 matched pairs. Matched pairs were randomized by one of the authors (MRL) using a computerized random number generator to either the CIS arm or the SOC arm using matched-pair randomization. Sequences were concealed until interventions were assigned. The study was non-blinded.

### Study population

Participants were enrolled in the SOC group beginning on April 22, 2013, and in the CIS group beginning on April 25, 2013. The last patient was enrolled in the SOC group on November 20, 2014, and the last patient in the CIS group was enrolled on February 11, 2015. Enrollment in the CIS+ group began after each clinic randomized to the intervention arm completed CIS enrollment, and ran from June 16, 2014, through June 30, 2015. All participants were followed for 12 months, with the last patient completing follow-up on June 30, 2016.

Broad inclusion criteria were used to reflect as accurately as possible the population of adults newly diagnosed with HIV in VCT clinics at the participating health facilities. We focused on individuals newly diagnosed in VCT clinics, as opposed to those diagnosed in antenatal clinics and tuberculosis clinics, because the latter groups of patients typically follow a modified clinic flow. All adults testing HIV positive in the VCT clinics within the participating health facilities were informed of the study by HIV testing counselors following diagnosis, and those who were interested were referred to study staff for further information, eligibility screening, and consent procedures. Patients were excluded if they were less than 18 years of age, were pregnant, planned to move from their community of residence in the next 12 months, had enrolled in HIV care or initiated ART in the past 6 months, did not understand Portuguese or Xitsua, or were incapable of providing informed consent. Study participants agreed to be referred to HIV care and treatment services at the same facility where they were diagnosed (referred to as the “diagnosing facility”); to complete a baseline, 1-month, and 12-month interview; to be traced at their homes if they could not be reached by phone for follow-up interviews; to provide contact information for a family member or friend who could provide information on their vital status if they could not be located for a follow-up interview; and, if they enrolled in HIV care and treatment services at the diagnosing facility, to have their clinical data abstracted from the facility’s existing electronic medical records.

### Study interventions

#### Standard of care

Participants at health facilities randomized to receive the SOC were managed as per prevailing Ministry of Health guidelines [27]. Individuals diagnosed with HIV received post-test counseling in the VCT clinic and were referred verbally to HIV services, typically in the diagnosing facility. Patients presenting to the facility receptionist to schedule a clinical consultation for HIV care were referred to the laboratory for CD4 cell count, chemistry, and hematology testing, and provided with an appointment 2–4 weeks later to allow sufficient time for the laboratory results to be received. ART eligibility was determined at that first clinical consultation based on CD4 cell count ≤ 350 cells/mm^3^ and/or WHO stage 3/4. Those found to be eligible for ART received at least 1 individual counseling session before initiating treatment. For ART-eligible patients, the time interval between enrollment in HIV care and ART initiation was estimated at 1–2 months at the time the study started. Participants initiating ART were requested to return every 2 weeks for the first month, at 2 months, at 6 months, and every 6 months thereafter. ART-ineligible patients were instructed to return at 6 months for repeat clinical evaluation and laboratory testing.

#### Combination intervention strategy

At facilities randomized to the intervention arm, we introduced 4 evidence-based interventions that simplified the clinic flow and encouraged linkage to and retention in care. These interventions targeted several known health system, structural, and behavioral barriers across the HIV care continuum, and were adapted for the on-the-ground realities—including practice norms, physical space, and available staffing—at the health facilities. First, we introduced Pima (Inverness Medical Innovations) CD4 assay machines in the VCT clinics to enable HIV testing counselors to provide real-time, point-of-care CD4 test results immediately following diagnosis, and thus addressed a health system barrier by reducing the number of visits required for CD4 testing. We also hypothesized that receipt of additional information on one’s health at the time of diagnosis would advance patient understanding of the need for care, a documented behavioral barrier [10,28]. All patients regardless of CD4 count were provided with a paper-based referral to on-site HIV services that included their CD4 count, and were instructed to present for their first clinical consultation within 1 week. Second, to address additional health system barriers, patients with Pima CD4 cell count ≤ 350 cells/mm^3^ were provided with accelerated ART initiation, with the ultimate goal of decreasing the HIV morbidity and mortality that contributes to significant attrition among ART-eligible patients [4]. These individuals received an individual ART preparatory counseling session in the VCT clinic immediately following CD4 testing, on the day of diagnosis. Facility receptionists were instructed to expedite appointments for these patients when they presented to schedule their clinical consultations. Although the patients were directed to the laboratory to have their blood drawn for baseline laboratory tests required by national ART guidelines, clinicians were encouraged to initiate ART at the first clinical visit rather than await the results of the laboratory tests unless the patient presented with comorbid conditions. Patients who initiated ART received a 2-week supply and followed the visit schedule dictated by national guidelines, similar to the SOC procedures. Once baseline laboratory results were available, they were reviewed by clinic staff, and if abnormalities were noted, the participant was contacted to return to the clinic. Third, participants received health messages and appointment reminders via SMS messaging to address behavioral barriers associated with deferring care engagement and forgetting appointments. The messages were sent from the central study office to the participant’s phone or to a friend or relative’s phone per participant preference, and did not refer to HIV or a specific health facility or reveal any personal information. The health messages encouraged participants to care for their health, and were sent weekly for 1 month following diagnosis and then monthly (e.g., “Hi. Your health is the most important thing. Please remember to come to the health center for health services.”). Appointment reminders were sent only to participants who linked to care at the diagnosing facility, and were sent 3–7 days before each scheduled clinic visit (e.g., “Hi. Your health is the most important thing. We expect to see you at your upcoming appointment scheduled for the day ___.”). Participants were not asked to confirm receipt or reply to the messages. Finally, patients in the CIS+ cohort received the CIS interventions plus a series of non-cash financial incentives (FIs) in the form of prepaid cellular air-time cards to offset structural barriers associated with the direct and indirect costs of coming to the health facility to receive HIV care. Air-time cards rather than cash were selected as the incentive based on discussion with the Ministry of Health. Each card was valued at approximately US$5 and was provided conditionally upon the following achievements: linkage to care within 1 month of diagnosis, retention in care 6 months after diagnosis, and retention in care 12 months after diagnosis, for a total of approximately US$15. Participants who completed each achievement received the card when presenting for routine services. Participants without cellular phones could opt to give them to a family member, sell them for cash, or trade them for other goods. Both the point-of-care CD4 testing and accelerated ART initiation interventions were provided by health facility staff to all individuals diagnosed with HIV in the VCT clinic regardless of whether they were enrolled in the study, while the SMS messages and FIs were provided by study staff and only to study participants.

### Data collection and outcomes

#### Site assessments

Data on the configuration of HIV services at the 10 participating study sites were collected at the beginning and at the end of the study using a standardized site assessment form. The purpose of the site assessments was to identify important similarities and differences between participating health facilities, as well as to better understand how services at the site could impact study implementation.

#### Baseline interview

Participants completed closed-ended questionnaires administered by trained research assistants at the time of study enrollment. The questionnaire took about 30 minutes to complete, and gathered information on sociodemographic characteristics, social and family support, mental health, alcohol use, HIV testing history, HIV knowledge and beliefs, and anticipated stigma and barriers to care. Anticipated stigma was assessed through 6 items adapted from the 12-item anticipated HIV stigma index developed by Earnshaw and Chaudoir [29]. Stigma scores were summed, then dichotomized into 2 groups: highest (>75th percentile) versus lower anticipated stigma. Mental health was assessed via a 7-question evaluation based on the Kessler 10-item scale for psychological distress [30]. Mental health scores were summed, then dichotomized into 2 groups: highest (<75th percentile) versus lower level of distress. Perceived availability of social support was assessed with 4 questions adapted from a 9-item scale by Wortman and colleagues [31]. Social support scores were summed, then dichotomized into 2 groups: higher (>50th percentile) versus lower social support. Questions assessing HIV-related knowledge and attitudes were based on those used by one of the authors in a previous study [32]. HIV knowledge scores were summed, then dichotomized into 2 groups: higher (>50th percentile) versus lower knowledge. Baseline interview data were double-entered into a study database, and a computer program identified discrepant double-entered results for correction against the paper-based forms.

#### Patient tracing and follow-up interviews

One and 12 months after enrollment, up until June 30, 2016, trained research assistants contacted participants by phone to ascertain their vital status and HIV care status, and to administer follow-up questionnaires. If the participant could not be contacted by phone after 3 attempts, research assistants visited the participant’s home up to 3 times. Participants who were located completed closed-ended interviews that gathered updated information on key domains from the baseline questionnaire, as well as self-reported information on linkage to (1- and 12-month questionnaires) and retention in HIV care (12-month questionnaire only), reasons for linkage/non-linkage (1- and 12-month questionnaires) and retention/non-retention (12-month questionnaire only), ART status, hospitalizations, and anticipated stigma. In cases where the participant could not be located, research assistants contacted a friend or family member as specified by the participant at study enrollment. Research assistants did not refer to HIV or the health facility during contact tracing but rather attempted to determine whether the participant was alive or dead. For those whose vital status could not be determined through contact tracing, research assistants searched existing electronic medical records at other primary health facilities supported by the Center for Collaboration in Health in the same district to assess whether patients had enrolled in HIV care at another facility, and reviewed death registers at the municipal and provincial levels to ascertain their vital status. Similar data entry and reconciliation procedures to those used for the baseline interview data were used for the tracing and follow-up data.

#### Abstraction of clinical data for patients linking to HIV care at the diagnosing facility

As part of routine clinical practice for HIV patients, clinicians documented patient information at every clinic visit on national HIV care forms, and trained data clerks entered those data into an Access-based electronic medical record. In its role as a PEPFAR implementing partner supporting the study sites, the Center for Collaboration in Health assessed the completeness and accuracy of these electronic data every 4 months and initiated targeted interventions to enhance data quality if there was greater than 15% disagreement on key data elements between the electronic and paper-based systems. During the study period, research assistants reviewed the electronic medical records to identify study participants who had linked to care at their diagnosing facility. For those located, we extracted the complete electronic medical record, capturing information on visit dates, vital status, transfer status, ART status, laboratory test results, and opportunistic infections.

#### Outcomes

The primary outcome was a combined outcome of linkage to HIV care within 1 month of diagnosis plus retention in care 12 months after diagnosis measured at the individual level. We used a combined outcome to reflect the fact that improvements are needed across the HIV care continuum in order to maximize individual and population health. Linkage to care was defined by at least 1 clinical consultation for HIV that included assessment of the patient’s medical history and a physical exam. Retention in care was defined by a clinic visit in the 90 days prior to the end of the 12-month study follow-up period, with no documentation that the patient had transferred to another facility or had died. We assessed the combined outcome from the perspective of the diagnosing health facility using data from the electronic medical records maintained by the HIV clinics. All study participants were included in these analyses, including those who did not complete follow-up interviews. Participants whose electronic medical records were not located were considered not to have achieved the combined outcome for this analysis. As a secondary approach, we evaluated the combined outcome from the perspective of the Mozambican health program by supplementing data from the electronic medical records with patient reports of linkage to and retention in care at HIV clinics at different health facilities (obtained during follow-up interviews) and information obtained from electronic medical records at other health facilities. In these analyses, participants whose self-reported linkage and retention status suggested they were linked to and/or retained at a health facility other than their diagnosing clinic were considered to have achieved the respective linkage/retention outcomes. Participants who either did not complete follow-up interviews or did not self-report linkage to or retention at another clinic maintained their initial outcome designation. All study participants were included in these analyses.

Secondary outcomes included linkage to care at several predefined time points, ART eligibility assessment (defined as receipt of WHO staging and/or CD4 cell count), ART initiation, disease progression (defined as a new WHO stage 3/4 condition or hospitalization noted in the electronic medical records or self-reported during follow-up interviews), retention in care 6 and 12 months after diagnosis regardless of the timing of linkage, and death.

### Statistical analysis

The trial was designed and powered to measure outcomes at the individual level, with outcomes assessed within each cluster (5 clusters per arm). In our initial power calculations, we anticipated that an average of 200 patients per clinic (in the CIS and SOC arms) would be eligible for enrollment based on historical data on the annual number of adults testing positive in the VCT clinics at the participating health facilities. With 5 facilities per study arm, an average of 200 patients per facility, an intraclass correlation coefficient (ICC) of 0.05, and an alpha of 0.05 and assuming that 35% of participants in the SOC arm would achieve the primary outcome, we estimated that the study would have 80% power to detect as statistically significant 55% of participants in the CIS group achieving the primary outcome, and greater than 80% power to detect as statistically significant 75% of participants in the CIS+ group achieving the primary outcome. Because enrollment proceeded slower than originally planned, at study midpoint we assessed the implications for power if each health facility enrolled an average of 150 participants rather than 200. Our calculations revealed minimal change in power with this reduction in the number of participants per health facility. Calculations were performed using PASS 8.0 software for 2 independent proportions in a cluster randomization study design and a 2-sided Farrington and Manning Likelihood Score Test [33]. Our power estimations and statistical analyses did not take into account the pair matching prior to randomization but rather followed recommendations from Diehr et al. [34] to break matches in statistical analyses of clustered studies when the number of pairs is between 3 and 9.

An intent-to-treat analysis determined the relative risk (RR) of achieving study outcomes between the CIS and SOC groups, and between the CIS+ and CIS groups. For analyses of the primary outcome, we used random-intercept multilevel log-Poisson models to account for clustering within health facilities with an empirical variance adjustment for small numbers of sampling units described by Morel et al. [35]. We also assessed whether the primary outcome differed after adjustment for patient-level factors by constructing propensity scores that estimated the probability of inclusion in the CIS, CIS+, and SOC groups by age, sex, region, education, income, employment status, marital status, religion, prior year history of being away from home for more than 1 month, travel time to clinic, tuberculosis status, past hospitalizations, diagnosis history, and whether another family member was known to be living with HIV. The propensity score was included as a covariate in the multivariable log-Poisson models (adjusted analyses). In post hoc analyses, we further estimated the likelihood of key subgroups achieving the primary outcome using interaction contrast ratios. The subgroups assessed included subgroups based on baseline age, sex, region of health facility, employment status, marital status, whether the participant was away from home for more than 1 month in the year prior to study enrollment, travel time to clinic, whether a household member was known to be HIV positive, and dichotomous variables based on scales for self-reported anticipated stigma, HIV knowledge, mental health, and perceived social support as described earlier. For analyses of secondary outcomes, log-Poisson models were used for dichotomous outcomes, and *t* tests and 2-way median tests as appropriate for continuous outcomes, adjusting for clustering but not for patient-level differences.

## Results

### Health facility characteristics

As noted above, 10 primary health facilities participated in the study, 4 in Maputo and 6 in Inhambane. At study start, the 5 health facilities randomized to the intervention arm reported that they had experienced disruptions of 3 or more days in VCT services in the prior 12 months, while only 1 facility randomized to the SOC arm reported experiencing a similar disruption. By study end, no facilities—whether in the intervention or SOC arm—had experienced such disruptions. Throughout the study, only intervention sites conducted point-of-care CD4 testing using Pima machines in the VCT clinic. Two SOC sites reported that they had Pima machines available in their laboratories but only used them to monitor CD4 counts after patients had enrolled in HIV care. None of the SOC sites used SMS messaging for health messages or appointment reminders on a routine basis for all patients, but 2 sites sent SMS appointment reminders for patients participating in community ART groups [36]. Though the 2013 national HIV treatment guidelines stipulate that 1 ART preparatory counseling session is required for ART-eligible patients, all the facilities participating in the study typically conducted 2 to 3 sessions prior to ART initiation, with a slight reduction in the number of sessions observed between study start and end.

### Enrollment and participant characteristics

**Fig 1:**
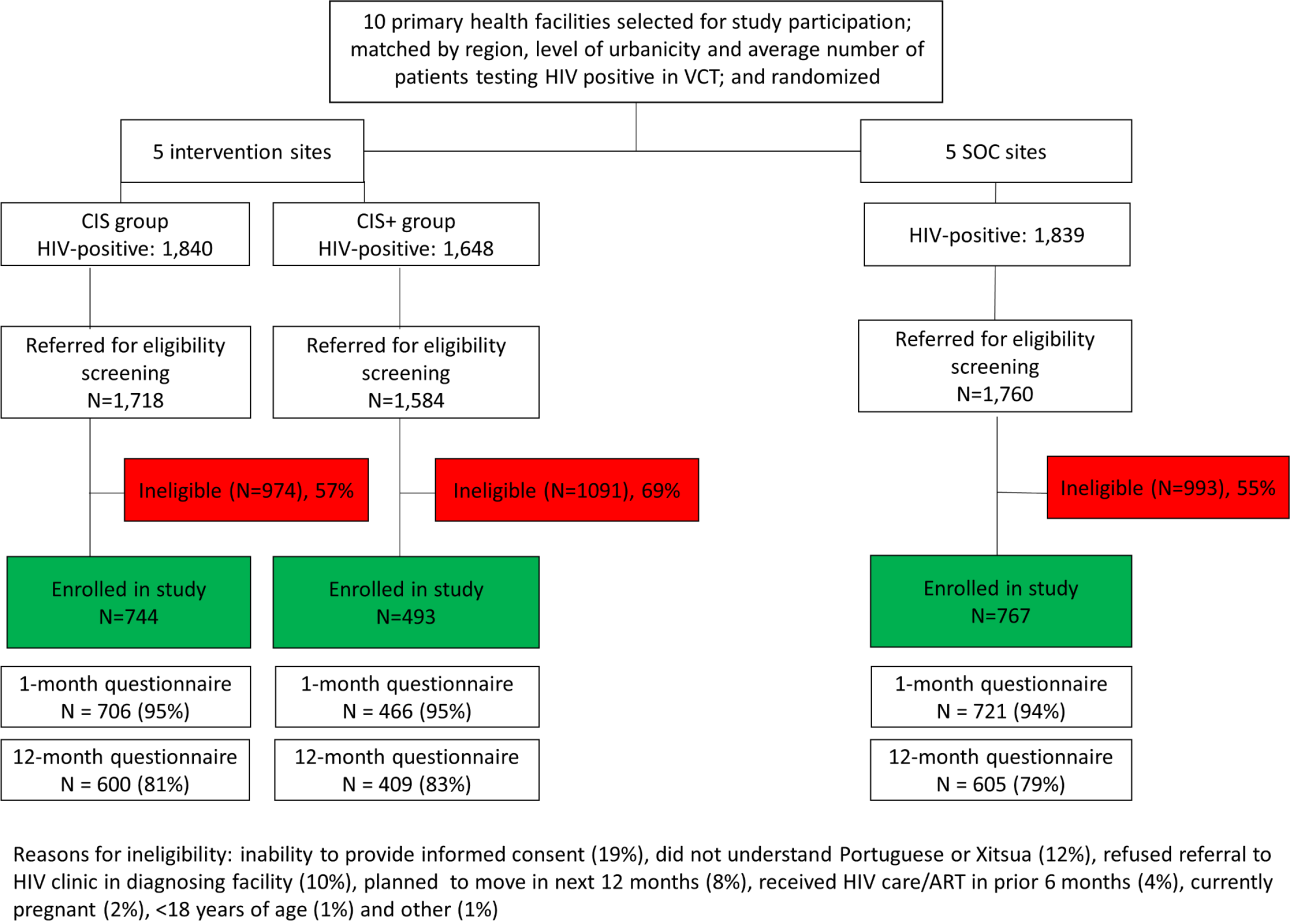
Flow chart for study participation. CIS, combination intervention strategy; SOC, standard of care; VCT, voluntary counseling and testing.

Fig 1 shows the enrollment, exclusion, and flow of the patients by study group. During the study period, 5,327 adults ≥18 years of age were diagnosed with HIV in the VCT clinics at the 10 study facilities. A total of 265 of those individuals were not referred to the study staff for further information on the study because they informed the HIV testing counselor that they were not interested in the study, were already receiving HIV services, or were not willing to be referred to the diagnosing health facility. Among the 5,062 who were referred to the study staff for further information, 3,058 did not meet study eligibility criteria. The main reasons for exclusion were inability to provide informed consent due to distress following diagnosis (19%), inability to understand Portuguese or Xitsua (12%), and refusal to be referred to the diagnosing health facility for HIV services (10%).

A total of 2,004 adults ≥18 years of age enrolled in the study at the 10 health facilities: 744 (37%) in the CIS group, 493 (25%) in the CIS+ group, and 767 (38%) in the SOC group. The majority of participants were female (64%), and the median age of participants was 34 years of age, with no meaningful differences observed by study group (Table 1). More than half of the participants (53%) were living with a partner at the time of diagnosis, and 65% of participants had a primary or lower level of education. Most participants (74%) were employed, and 43% had a monthly income of less than 1,500 meticais (approximately US$50). One-quarter (27%) reported that another household member was living with HIV. While no serious adverse events were reported during the study period, there was 1 unanticipated event of a female participant reporting intimate partner violence. The Mozambican National Committee for Bioethics for Health and the Columbia University IRB were informed of this event, and the participant asked to remain in the study but to conduct all study interviews at the facility (i.e., no follow-up phone calls).

**Table 1:** Participant characteristics at study enrollment in the 3 study groups (*N =* 2,004).

Characteristic	Total *N =* 2,004	CIS *N =* 744	CIS+ *N =* 493	SOC *N =* 767	*p-*Value
**Region**					
Maputo	1,077 (54%)	396 (53%)	275 (56%)	406 (53%)	0.58
Inhambane	927 (46%)	348 (47%)	218 (44%)	361 (47%)	
**Sex**					0.50
Female	1,292 (64%)	490 (66%)	319 (65%)	483 (63%)	
Male	712 (36%)	254 (34%)	174 (35%)	284 (37%)	
**Age (years)**	34.2 (9.6)	34.9 (9.8)	33.8 (9.9)	33.8 (9.3)	0.045
18–24	265 (13%)	90 (12%)	70 (14%)	105 (14%)	0.12
25–39	1,233 (62%)	440 (59%)	301 (61%)	492 (64%)	
40–49	348 (17%)	148 (2%)	87 (18%)	113 (15%)	
50+	158 (8%)	66 (9%)	35 (7%)	57 (7%)	
**Marital status**					<0.001
Married/partner and living together	1,068 (53%)	376 (51%)	255 (52%)	437 (57%)	
Married/partner, but not living together	222 (11%)	101 (14%)	86 (17%)	35 (5%)	
Single	713 (36%)	266 (36%)	152 (31%)	295 (38%)	
Missing/refused	1 (0%)	1 (0%)	0 (0%)	0 (0%)	
**Education**					0.003
None	164 (8%)	59 (8%)	33 (7%)	72 (9%)	
Primary	1,149 (57%)	442 (59%)	256 (52%)	451 (59%)	
Secondary	471 (24%)	164 (22%)	130 (26%)	177 (23%)	
Above secondary	219 (11%)	78 (1%)	74 (15%)	67 (9%)	
Missing/refused	1 (0%)	1 (0%)	0 (0%)	0 (9%)	
**Employment**					0.46
Employed	1,473 (74%)	537 (72%)	361 (73%)	575 (75%)	
Unemployed	531 (26%)	207 (28%)	132 (27%)	192 (25%)	
**Monthly income**					<0.001
≤1,500 meticais	871 (43%)	342 (46%)	165 (33%)	364 (47%)	
>1,500 meticais	936 (47%)	343 (46%)	271 (55%)	322 (42%)	
Missing/refused	197 (1%)	59 (8%)	57 (12%)	81 (11%)	
**Another household member has HIV**					0.28
Yes	550 (27%)	187 (25%)	144 (29%)	219 (29%)	
No	913 (46%)	361 (49%)	219 (44%)	333 (43%)	
Don’t know	539 (27%)	196 (26%)	130 (26%)	213 (28%)	
Missing/refused	2 (0%)	0 (0%)	0 (0%)	2 (0%)	

Data given as *N* (percent).

CIS, combination intervention strategy; SOC, standard of care.

### Intervention effect on linkage to and retention in HIV care at the diagnosing facility

**Fig 2:**
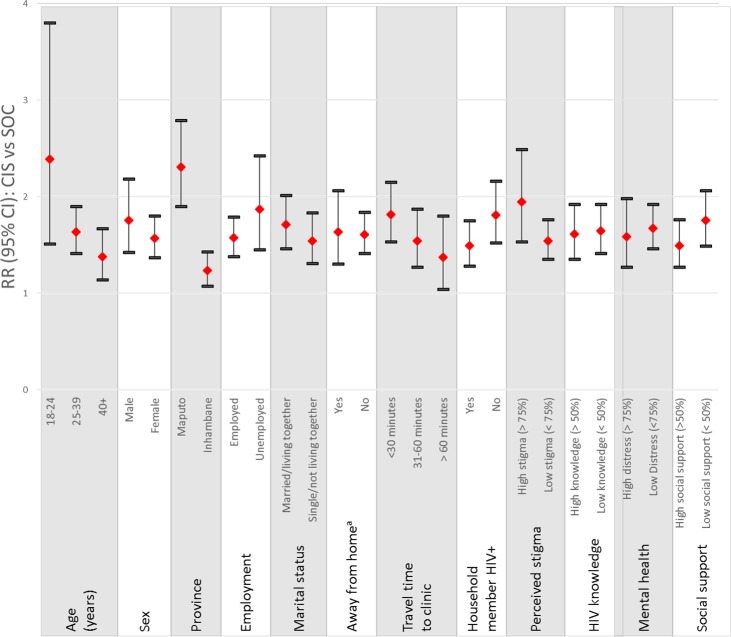
Relative risk of the CIS compared to the SOC on the primary outcome at the diagnosing health facility by patient characteristics. ^a^ Fifteen patients with missing information were excluded from this estimate. A description of the variables examined and categories used are provided in the Methods section.

As shown in Table 2, the CIS was associated with statistically significant improvements in the combined outcome of linkage to care within 1 month of diagnosis and retention in care 12 months following diagnosis when compared to the SOC. Analyses using data from electronic medical records to examine linkage to and retention at the diagnosing health facility showed that 57% of participants in the CIS group achieved the primary outcome versus 35% of those in the SOC group (RR_CIS vs SOC_ = 1.58, 95% CI 1.05–2.39). Post hoc calculation of the ICC for the primary outcome according to the methods of Snijders and Bosker for binary outcome data [37] estimated an ICC of 0.066, similar to but slightly higher than the assumed ICC of 0.05 used in power and sample size estimation. These results were robust to adjustment for patient-level differences (adjusted RR [aRR]_CIS vs SOC_ = 1.55, 95% CI 1.07–2.25). As shown in Fig 2, the greatest intervention effects were observed among young adults age 18–24 years (RR_CIS vs SOC_ = 2.39, 95% CI 1.51–3.80, *p-*value for interaction between age and treatment arm = 0.07), those in Maputo (RR_CIS vs SOC_ = 2.31, 95% CI 1.90–2.79, *p-*value for interaction between region and treatment arm < 0.0001), those who did not report that another household member was living with HIV (RR_CIS vs SOC_ = 1.81: 95% CI 1.52–2.16, *p-*value for interaction between household member with HIV and treatment arm = 0.11), and those reporting high levels of anticipated stigma at enrollment (RR_CIS vs SOC_ = 1.95, 95% CI 1.53–2.49, *p-*value for interaction between stigma and treatment arm = 0.10).

**Table 2:** Linkage to and retention in HIV care: CIS versus SOC and CIS+ versus CIS.

Category	Outcome	CIS *N =* 744		CIS+ *N =* 493		SOC *N =* 767		RR^1^ (95% CI), *p*-Value		aRR^2^ (95% CI), *p*-Value	
		*N*	Percent	*N*	Percent	*N*	Percent	CIS versus SOC	CIS+ versus CIS	CIS versus SOC	CIS+ versus CIS
**Primary outcome**											
At diagnosing facility	Linked to care within 1 month of diagnosis and retained 12 months after diagnosis	425	57%	273	55%	268	35%	1.58 (1.05–2.39) *p* = 0.03	0.96 (0.81–1.16) *p* = 0.66	1.55 (1.07–2.25) *p* = 0.04	0.94 (0.76–1.18) *p* = 0.52
At any health facility	Linked to care within 1 month of diagnosis and retained 12 months after diagnosis	547	74%	360	73%	363	47%	1.47 (1.08–2.01) *p* = 0.02	0.98 (0.85–1.15) *p* = 0.91	1.46 (1.05–2.04) *p* = 0.03	0.96 (0.83–1.11) *p* = 0.52
**Secondary outcomes**											
Linkage at diagnosing facility	Same day as HIV test	659	89%	457	93%	120	16%	9.13 (1.65–50.40) *p* = 0.02	1.04 (0.92–1.20) *p* = 0.38	N/A	
	Within 1 week of HIV test	678	91%	461	94%	349	46%	2.43 (0.70–8.41) *p* = 0.14	1.03 (0.91–1.16) *p* = 0.59	N/A	
	Within 1 month of HIV test	703	94%	467	95%	482	63%	1.48 (0.93–2.35) *p* = 0.09	1.00 (0.89–1.13) *p* = 0.96	N/A	
	Within 12 months of HIV test	716	96%	467	95%	592	77%	1.23 (1.03–1.48) *p* = 0.03	0.98 (0.87–1.11) *p* = 0,74	N/A	
Retention at diagnosing facility	6 months after diagnosis	462	62%	322	65%	405	53%	1.18 (1.00–1.39) *p* = 0.06	1.05 (0.88–1.26) *p* = 0.48	N/A	
	12 months after diagnosis	435	58%	273	55%	341	44%	1.32 (1.12–1.54) *p* = 0.004	0.95 (0.79–1.13) *p* = 0.45	N/A	

^1^RR accounts for clustering within sites using random-intercept log-Poisson regression with empirical standard error estimates.

^2^aRR adjusts for patient-level differences using propensity scores.

aRR, adjusted relative risk; CIS, combination intervention strategy; N/A, not applicable; RR, relative risk; SOC, standard of care.

Eighty-nine percent of participants in the CIS group linked to the diagnosing facility on the same day as diagnosis compared to 16% (RR_CIS vs SOC_ = 9.13, 95% CI 1.65–50.40) in the SOC group, 91% within 1 week compared to 46% (RR_CIS vs SOC_ = 2.43, 95% CI 0.70–8.41), and 94% within 1 month compared to 63% (RR_CIS vs SOC_ = 1.48, 95% CI 0.93–2.35). By 12 months, nearly all CIS participants (96%) had linked to care compared to 77% (RR_CIS vs SOC_ = 1.23, 95% CI 1.03–1.48) of SOC participants. Among those linking to care, the median (interquartile range [IQR]) time from diagnosis to linkage was 0 days (0–0) in the CIS group and 3 days (1–26) in the SOC group (median test *p* < 0.001 for CIS versus SOC). The effect of the intervention on retention in care, regardless of the timing of linkage, was more modest but statistically significant (6-month retention: 62% CIS versus 53% SOC, RR_CIS vs SOC_ = 1.18, 95% CI 1.00–1.39; 12-month retention: 58% CIS versus 44% SOC, RR_CIS vs SOC_ = 1.32, 95% CI 1.12–1.54).

In analyses restricted to the participants initiating ART, the median (IQR) time from diagnosis to ART initiation in the CIS and SOC groups was 32 (12–135), and 63 (33–230) days, respectively, while the median (IQR) time from enrollment in HIV care to ART initiation was 32 (11–127), and 50 (15–205) days, respectively. Median time from ART eligibility to ART initiation for the CIS, CIS+, and SOC groups was 21 (9–40), and 25 (11–56) days, respectively.

There was no additional benefit of adding FIs to the CIS, with 55% (RR_CIS+ vs CIS_ = 0.96, 95% CI 0.81–1.16; aRR_CIS+ vs CIS_ = 0.94_,_ 95% CI 0.76–1.18) of those in the CIS+ group achieving the primary outcome; 95% (RR_CIS+ vs CIS_ = 1.00, 95% CI 0.83–1.13) linking to HIV care within 1 month of diagnosis, regardless of retention at 12 months; and 55% (RR_CIS+ vs CIS_ = 0.95, 95% CI 0.79–1.13) being retained in care 12 months after diagnosis, regardless of the timing of linkage to care.

### Intervention effect on linkage to and retention in care at any health facility

Analyses supplementing data from electronic medical records from participating facilities with data from patient interviews and other health facilities in the study regions to examine linkage to and retention at any health facility showed similar effects of the intervention package. A total of 74% (RR_CIS vs SOC_ = 1.47, 95% CI 1.08–2.01) of participants in the CIS group and 47% in the SOC group were found to have linked to HIV care at any health facility within 1 month of diagnosis and were retained in HIV care 12 months after diagnosis (Table 2). Adjustment for patient-level differences did not result in any change in this finding (aRR_CIS vs SOC_ = 1.46, 95% CI 1.05–2.04). Inclusion of FIs in the CIS also showed no additional benefit for linkage to and retention at any health facility, with 73% (RR_CIS+ vs CIS_ = 0.98, 95% CI 0.85–1.15; aRR_CIS+ vs CIS_ = 0.96, 95% CI 0.83–1.11) of those in the CIS+ group known to have linked to and been retained in HIV care at any health facility compared to the CIS group.

### Intervention effect on ART eligibility and initiation, disease progression, and death

Data from electronic medical records at study sites indicated that compared to patients in the SOC group, patients in the CIS group were more likely to ever have their ART eligibility assessed (100% versus 76.9%, RR_CIS vs SOC_ = 1.29, 95% CI 1.08–1.54), be identified as ART eligible (75% versus 60%, RR_CIS vs SOC_ = 1.24, 95% CI 1.07–1.43), and initiate ART (65% versus 54%, RR_CIS vs SOC_ = 1.20, 95% CI 1.00–1.43) (Table 3). Very few participants were diagnosed with a new WHO stage 3/4 event at the diagnosing facility or self-reported a hospitalization in the 12 months after HIV diagnosis. Those in the CIS group had a non-significantly but modestly decreased risk compared to those in the SOC group (1% versus 3%, RR_CIS vs SOC_ = 0.38, 95% CI 0.07–2.03), while similar results were observed between the CIS and CIS+ groups (1% versus 1%, RR_CIS+ vs CIS_ = 0.65, 95% CI 0.12–3.64). Neither the CIS nor the CIS+ interventions had a significant effect on mortality within 12 months of diagnosis, with 6%, 5%, and 7% of participants in the CIS, CIS+, and SOC groups, respectively, known to have died during study follow-up (RR_CIS vs SOC_ = 0.87, 95% CI 0.40–1.91; RR_CIS+ vs CIS_ = 0.88, 95% CI 0.45–1.74). The CIS also did not have a significant impact on mortality before (3%, RR_CIS vs SOC_ = 0.78, 95% CI 0.46–1.32) or after ART initiation (3%, RR_CIS vs SOC_ = 0.96, 95% CI 0.26–3.48); participants in the CIS+ group were less likely to die, though non-significantly so, before initiating ART compared to those in the CIS group (1% versus 3%, RR_CIS+ vs CIS_ = 0.34, 95% CI 0.09–1.29).

**Table 3:** ART determination and initiation, disease progression, and death: CIS versus SOC and CIS+ versus CIS.

Outcome	CIS (*N =* 744)		CIS+ (*N =* 493)		SOC (*N =* 767)		RR^1^ (95% CI), *p*-value	
	*N*	Percent	*N*	Percent	*N*	Percent	CIS versus SOC^1^	CIS+ versus CIS^1^
ART eligibility assessed	744	100%	493	100%	590	77%	1.29 (1.08–1.54) *p* = 0.01	1.00 (0.89–1.12) *p* = 1.00
Identified as ART eligible	557	75%	372	75%	464	60%	1.24 (1.07–1.43) *p* = 0.01	1.01 (0.85–1.19) *p* = 0.91
Initiated ART	484	65%	332	67%	416	54%	1.20 (1.00–1.43) *p* = 0.05	1.03 (0.88–1.22) *p* = 0.59
New WHO stage 3/4 or hospitalization	7	1%	3	1%	23	3%	0.38 (0.07–2.03) *p* = 0.22	0.65 (0.12–3.64) *p* = 0.53
Death within 12 months	46	6%	27	5%	54	7%	0.87 (0.40–1.91) *p* = 0.69	0.88 (0.45–1.74) *p* = 0.63
Death before ART initiation	22	3%	5	1%	29	4%	0.78 (0.46–1.32) *p* = 0.31	0.34 (0.09–1.29) *p* = 0.09
Death after ART initiation	24	3%	22	4%	25	3%	0.96 (0.26–3.48) *p* = 0.94	1.38 (0.62–3.07) *p* = 0.33

^1^RR accounts for clustering within sites using random-intercept log-Poisson regression with empirical standard error estimates.

ART, antiretroviral therapy; CIS, combination intervention strategy; RR, relative risk; SOC, standard of care.

## Discussion

We conducted a cluster-randomized study in Mozambique to examine the effectiveness of a multi-component approach to increase linkage to and retention in HIV care—2 critical elements of the HIV care continuum—among adults newly diagnosed with HIV. The operational model of the CIS that we evaluated addresses known structural, biomedical, and behavioral barriers across the HIV care continuum and was composed of evidence-based, practical, and scalable interventions, including CD4 testing in VCT clinics with immediate turnaround of results, accelerated ART initiation for eligible individuals, and SMS health messages and appointment reminders. An enhanced version of the CIS additionally included FIs. In the spirit of implementation science, 2 of the interventions were implemented by existing health facility staff, rather than study staff, providing information on the real-world successes and challenges associated with the CIS that can be extrapolated to a range of settings with similar implementation contexts.

Our study showed that participants receiving the CIS were 1.58 times more likely to link to HIV care at their diagnosing facility within 1 month of diagnosis and be retained in care at that same facility 12 months following diagnosis, representing not only a statistically significant but also a programmatically meaningful improvement. Particularly impressive gains were observed in timely linkage to care at the diagnosing facility: 89% of CIS participants linked to care on the day of diagnosis, representing a greater than 5-fold improvement compared to the SOC, and nearly universal linkage (96%) was achieved within 1 month of diagnosis. Notably, the intervention effect was greatest in subpopulations documented to have particularly poor outcomes across the HIV care continuum, including young adults [38,39] and those with high stigma perceptions [40–42]. The intervention also had beneficial effects on other important milestones in the HIV care continuum in the 12 months following diagnosis, including the likelihood of patients having their ART eligibility assessed and initiating ART. While the intervention significantly increased retention in HIV care at both 6 and 12 months following diagnosis, retention in the CIS group remained concerningly low and far short of what is needed to end the HIV epidemic in Mozambique and other high-burden countries.

We found no additional gain in effectiveness from adding FIs to the CIS. Prior studies examining the effect of FIs in enhancing outcomes across the HIV care continuum among people living with HIV have shown inconsistent results. Studies from India, Uganda, and Democratic Republic of the Congo reported reductions in time to ART initiation and improvements in retention with the provision of incentives, while in the United States, randomized trials did not show any effect of FIs on linkage to care or viral load suppression [43–47]. While 89% of participants in the current study reported that the type of FI provided and the amount of the FIs (i.e., mobile phone air-time vouchers worth approximately US$5 at 3 points in time) were adequate, it is possible that the FIs were not sufficiently optimized to affect behaviors. Indeed, as reported elsewhere, patient reactions to the FIs were surprisingly tepid, with only 21% reporting it to be the “most useful” intervention for retention in care 12 months following diagnosis [21]. Additionally, fidelity to the FI component of the intervention package was imperfect, with, for example, 86% of participants eligible to receive the first incentive actually receiving it, which may have further limited the effect of this intervention [21]. However, given the benefits of FIs in other health sectors [48–50], further research is needed to understand whether and how they may be optimized to enhance outcomes across the HIV care continuum.

This study has several important strengths. It is among the first studies to evaluate the impact of a multi-component approach on 2 important HIV care and treatment indicators: timely linkage to care following an HIV diagnosis and sustained retention in care. Improving performance for these 2 elements of the HIV care continuum is critical for realizing the individual and population benefits of HIV programming in sub-Saharan Africa. Further, while studies have examined the effectiveness of multi-component intervention packages that include FIs on HIV care outcomes [51,52], this study is the first to our knowledge to use a design that permits estimation of the additional benefit of including FIs as part of such a package.

Our study also had limitations. First, in alignment with recent recommendations for implementation science studies [19], we used existing electronic medical records in the HIV clinics at the study sites to ascertain outcomes at the diagnosing facility, but such records may have limited data quality. However, data quality assessments were conducted regularly during the study period and ensured at least 85% concurrence between paper-based and electronic medical records on key data elements. Second, aside from the FI, we cannot unpack the effect of individual intervention components. Third, the relevance of point-of-care CD4 count testing may change as countries adopt “treatment for all” strategies, although our results suggest that providing people living with HIV with additional information on their health status immediately following diagnosis may be important in facilitating same-day linkage to care and likely same-day ART initiation. Fourth, the CIS+ cohort was enrolled once the target sample size had been reached in the CIS cohort, thus introducing the potential for secular trends to have biased the comparison of the CIS and CIS+ packages. However, because we found no difference in the primary outcome between the CIS+ and CIS groups, secular trends would have had to have operated in the direction of reducing overall linkage and retention for this bias to result in the failure to observe an additional benefit of FIs for linkage and retention. While this is plausible, we do not have any evidence that a substantial reduction in overall linkage and retention occurred over the relatively limited time frame of the study. Finally, while the study was implemented in 2 contrasting settings within Mozambique, study facilities were located primarily in urban and semi-urban areas within the city of Maputo and Inhambane Province, which may limit generalizability. Indeed, settings with lower education and cell phone coverage than those included in our study may experience greater challenges implementing the SMS health messages and appointment reminders. Similarly, while we set broad inclusion criteria, we did exclude people who did not understand Portuguese or Xitsua, were planning on leaving the community, or were not willing to receive services at the diagnosing facility, all factors that may have reduced generalizability. Finally, due to slower-than-expected enrollment, we enrolled fewer participants in the CIS+ group than intended, which decreased our power to detect statistically significant differences in study outcomes between the CIS+ and CIS groups. However, as the proportion achieving the combined outcome in the 2 groups was extremely similar (CIS 57% versus CIS+ 55%), it is unlikely that the inability to detect significant differences was primarily due to lack of power.

## Conclusion

Multi-component intervention strategies have been proposed to address troubling gaps in the HIV care continuum [17,18]. To our knowledge, this is amongst the first studies to rigorously evaluate such an approach. The CIS we examined, comprising 3 evidence-based, practical, and scalable interventions, holds great promise as an approach to make much needed gains in the HIV care continuum in sub-Saharan Africa, particularly in the critical first step of timely linkage to care following diagnosis.

## Supporting information

S1 Text Study protocol. (PDF)

S2 Text CONSORT checklist. (DOCX)

S1 Data Data file. (CSV)

S2 Data Data codebook. (XLSX)

## Abbreviations

aRR: adjusted relative risk
ART: antiretroviral therapy
CIS: combination intervention strategy
FI: financial incentive
ICC: intraclass correlation coefficient
IQR: interquartile range
IRB: institutional review board
RR: relative risk
SMS: short message service
SOC: standard of care
VCT: voluntary counseling and testing
