# Supplementary material for: A combination intervention strategy to improve linkage to and retention in HIV care following diagnosis in Mozambique: A cluster-randomized study
Source: PLoS Med. 2017 Nov 14;14(11):e1002433. doi: 10.1371/journal.pmed.1002433 (PMC5685437; doi:10.1371/journal.pmed.1002433)
Supplement: S1 Text — (PDF) [file pmed.1002433.s001.pdf]

Elul et al. BMC Infectious Diseases 2014, 14:549
http://www.biomedcentral.com/1471-2334/14/549

BMC Infectious Diseases

STUDY PROTOCOL Open Access

A combination strategy for enhancing linkage to and retention in HIV care among adults newly diagnosed with HIV in Mozambique: study protocol for a site-randomized implementation science study

Batya Elul[1,2*], Maria Lahuerta[1,2], Fatima Abacassamo[3], Matthew R Lamb[1,2], Laurence Ahoua[4], Margaret L McNairy[1], Maria Tomo[3], Deborah Horowitz[5], Roberta Sutton[1], Antonio Mussa[4], Danielle Gurr[1] and Ilesh Jani[6]

Abstract

Background: Despite the extraordinary scale up of HIV prevention, care and treatment services in sub-Saharan Africa (SSA) over the past decade, the overall effectiveness of HIV programs has been significantly hindered by high levels of attrition across the HIV care continuum. Data from "real-life" settings are needed on the effectiveness of an easy to deliver package of services that can improve overall performance of the HIV care continuum.

Methods/Design: We are conducting an implementation science study using a two-arm cluster site-randomized design to determine the effectiveness of a combination intervention strategy (CIS) using feasible, evidence-based, and practical interventions—including (1) point-of-care (POC) CD4 count testing, (2) accelerated antiretroviral therapy initiation for eligible individuals, and (3) SMS reminders for linkage to and retention in care—as compared to the standard of care (SOC) in Mozambique in improving linkage and retention among adults following HIV diagnosis. A pre-post intervention two-sample design is nested within the CIS arm to assess the incremental effectiveness of the CIS plus financial incentives (CIS + FI) compared to the CIS without FI on study outcomes. Randomization is done at the level of the study site, defined as a primary health facility. Five sites are included from the City of Maputo and five from Inhambane Province. Target enrollment is a total of 2,250 adults: 750 in the SOC arm, 750 in the CIS cohort of the intervention arm and 750 in the CIS + FI cohort of the intervention arm (average of 150 participants per site). Participants are followed for 12 months from time of HIV testing to ascertain a combined endpoint of linkage to care within 1 month after testing and retention in care 12 months from HIV test. Cost-effectiveness analyses of CIS compared to SOC and CIS + FI compared to CIS will also be conducted.

Discussion: Study findings will provide evidence on the effectiveness of a CIS and the incremental effectiveness of a CIS + FI in a "real-life" service delivery system in a SSA country severely impacted by HIV.

Trial registration: Clinicaltrials.gov, NCT01930084

Keywords: Linkage, Retention, ART, Cluster randomized trial, Implementation science

* Correspondence: be2124@cumc.columbia.edu
[1]ICAP, Columbia University, New York, NY, USA
[2]Department of Epidemiology, Mailman School of Public Health, New York, NY, USA
Full list of author information is available at the end of the article

BioMed Central

© 2014 Elul et al.; licensee BioMed Central Ltd. This is an Open Access article distributed under the terms of the Creative Commons Attribution License (http://creativecommons.org/licenses/by/4.0), which permits unrestricted use, distribution, and reproduction in any medium, provided the original work is properly credited. The Creative Commons Public Domain Dedication waiver (http://creativecommons.org/publicdomain/zero/1.0/) applies to the data made available in this article, unless otherwise stated.

Background

Although the extraordinary scale-up of HIV testing, care, and treatment programs in sub-Saharan Africa (SSA) has led to more than eight million persons initiating antiretroviral therapy (ART), the overall effectiveness of HIV programs has been significantly hindered by high levels of attrition across the HIV care continuum in the region [1-4]. Linkage to HIV care is the first step in the HIV care continuum and is essential for assessing the health of patients, determining ART eligibility and promptly initiating ART among eligible patients (Figure 1) [5]. Systematic reviews of studies in SSA indicate that on average 59-78% of adults testing HIV positive link to care within three months of diagnosis, as determined by receipt of a CD4 count assessment [3,6]. Retention in care, another critical step in the HIV care continuum, is required for prevention and treatment of opportunistic infections, continued clinical and laboratory monitoring for ART eligibility among those not eligible upon enrollment in care, timely ART initiation, and follow-up and provision of supportive care [7-14]. However, recent reviews from SSA estimate that only 45% of patients not yet eligible to initiate ART are retained in pre-ART care, only 66% of eligible individuals initiate ART, and of those initiating, only 65% are retained after three years [6,15]. Ultimately, less than one-third of individuals testing HIV positive in SSA are believed to have linked to and engaged in care 12 months after diagnosis [8,9].

Interventions to improve linkage to and retention in care can target structural, biomedical, and/or behavioral barriers reported by patients [5]. Reported structural barriers include the multiple visits required for counseling and ART eligibility determination; transport costs and distances; and financial, work, and childcare constraints [16-21]. Interventions addressing these barriers include point of care (POC) CD4 count testing immediately following diagnosis to consolidate laboratory testing to determine ART eligibility at the time of HIV testing, and financial incentives (FI) to offset the financial costs incurred through lost wages, transport, and other costs of care [22-25]. Biomedical barriers include delayed ART initiation among eligible patients, which may be remediated through accelerated or 'fast-track' ART initiation. Behavioral barriers include patients forgetting appointments and lack of understanding of HIV disease and associated care, including the need for care while healthy, which can be addressed through appointment reminders.

Mozambique, a country in Southern Africa severely affected by the HIV epidemic, has an HIV prevalence of 11.5% [26] and an estimated 1.7 million people living with HIV (PLWH) [27]. The Government of Mozambique has made considerable efforts to strengthen its health system to address the HIV epidemic such that by December 2012, more than 500,000 patients were enrolled in HIV care and treatment services, and 300,000 PLWH had initiated ART [28]. Despite these achievements, several important challenges remain in ensuring high quality care across the HIV care continuum in Mozambique. First, linkage to care following HIV diagnosis in Mozambique is believed to be very low with the limited available data indicating that only 57% of adults newly diagnosed with HIV enroll in care within 30 days after diagnosis [29]. Further, for individuals who do enroll in HIV care, retention is among the lowest in SSA, with recent data indicating that less than 50% of patients not yet eligible for ART remain in care at 12 months and less than 50% of patients on ART are retained 12 months later [29-33].

In order to address these challenges, we are conducting an implementation science study assessing the effectiveness and cost-effectiveness of a combination intervention strategy (CIS) comprised of several feasible, evidence-based and practical interventions on a combined outcome of linkage to and retention in comprehensive care among adults newly testing HIV positive in Mozambique.

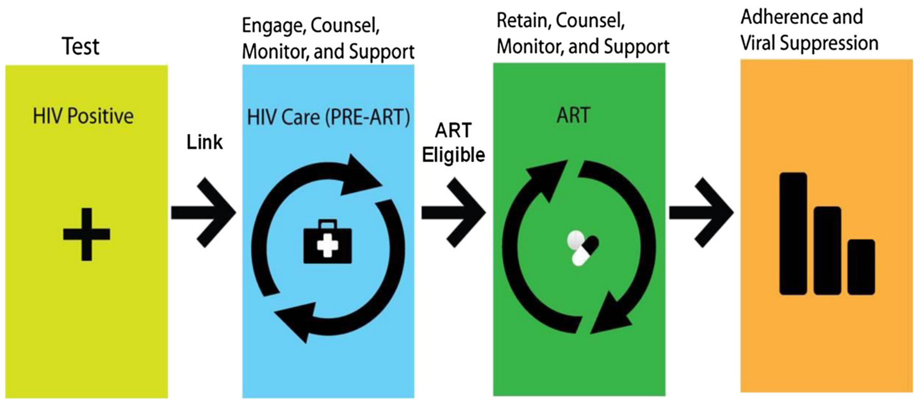

Figure 1 The HIV care continuum [5].

Primary objective

The primary objective is to evaluate the effectiveness of CIS compared to SOC, and of CIS with the addition of financial incentives (CIS + FI) compared to CIS, on the combined outcome of linkage to HIV care within 1 month and retention in care at 12 months at the same health facility of diagnosis among adults newly testing HIV positive.

Secondary objectives

The secondary objectives include to: 1) evaluate the effectiveness of CIS compared to SOC and the incremental effectiveness of CIS + FI compared to CIS in distinct steps along the HIV care continuum, including linkage to HIV care within 12 months from positive HIV test, retention in care at 12 months from positive HIV test, time from ART eligibility to ART initiation, and disease progression (new World Health Organization [WHO] stage III/IV event, hospitalization, CD4 count, death); 2) evaluate the cost-effectiveness of CIS compared to SOC and the incremental cost-effectiveness of CIS + FI compared to CIS; and 3) assess feasibility and participant acceptability of the relative benefit of the interventions included in CIS and CIS + FI in enhancing linkage and retention.

Methods

Study design

The study uses a two-arm cluster site-randomized design. Additionally, a pre-post intervention two-sample design is nested within the intervention arm to assess the incremental effectiveness of CIS + FI compared to CIS without FI on study outcomes. One cohort of participants (cohort 1) is enrolled in the SOC arm and two cohorts (cohorts 2 and 3) are enrolled sequentially in the intervention arm (Figure 2). Cohort 2 receives CIS consisting of POC CD4 count testing at time of HIV testing, accelerated ART initiation, and cellular appointment reminders and is enrolled concurrently to cohort 1 in the SOC arm; outcomes among cohort 2 will be compared to those among cohort 1. Cohort 3 receives CIS + FI, consisting of CIS plus FI at key points in the HIV care continuum and is enrolled following enrollment of all participants in cohort 2 in the CIS arm; outcomes among cohort 3 will be compared to those among cohort 2.

Study sites and participants

The study site is defined as a primary health facility providing both HIV testing and HIV care and treatment services. Within a given health facility, all HIV testing and counseling (HCT) points are included with the exception of prevention of mother-to-child transmission (PMTCT) and Tuberculosis (TB) clinic points of testing, as these testing points follow different linkage to care procedures. Primary health facilities were chosen as study sites, rather than larger provincial hospitals, as they reflect the decentralized HIV care system in Mozambique in which HIV services are provided in an outpatient clinic setting. A total of 10 sites were selected from the 66 health facilities in the region based on existing data suggesting they had sufficient numbers of patients testing HIV positive and enrolling in HIV care to meet the study sample size needs (see below) (Table 1). Sites are randomized to the SOC arm or the intervention arm using matched-pair randomization with matching done by facility location (rural, peri-urban, or urban) and expected number of adults enrolling in HIV care at each facility per month.

The target study population includes 2,250 treatment-naïve adults who newly test HIV positive at study sites: 750 in the SOC arm (cohort 1), 750 in the CIS cohort of the intervention arm (cohort 2) and 750 in the CIS + FI cohort of the intervention arm (cohort 3), with an average of 150 participants per site. Study participants must be 18 years of age or older, test positive at the participating HCT point within the health facility with written proof of test result, agree to be referred to HIV care services within

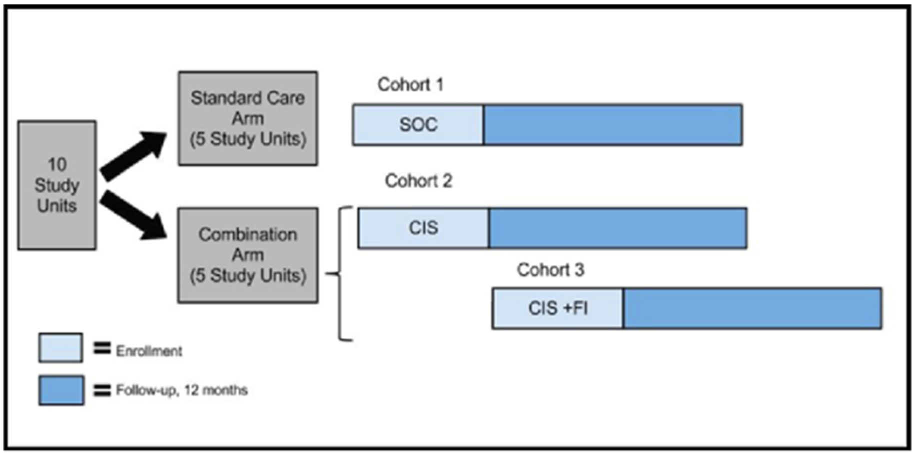

Figure 2 Overview of study design.

Table 1 Study sites, randomization assignments, and HIV counseling and testing points

Matched pair	Province	Facility	Randomization assignment	Urban/Rural locale	HIV testing point for study enrollment	Number of enrollees in HIV care
1	Maputo City	Bagamoio	CIS	Urban	VCT	>170 per month
	Maputo City	Mavalane	SOC	Urban	PICT*	>170 per month
2	Inhambane	Urbano	CIS	Urban	VCT	< 170 per month
	Inhambane	Maxixe	SOC	Urban	VCT	< 170 per month
3	Maputo City	Jose Macamo	SOC	Peri-urban	VCT	>170 per month
	Maputo City	Zimpeto	CIS	Peri-urban	VCT	>170 per month
4	Inhambane	Massinga	SOC	Peri-urban	VCT	< 170 per month
	Inhambane	Chicuque	CIS	Peri-urban	VCT	< 170 per month
5	Inhambane	Morrumbene	SOC	Rural	PICT**	< 170 per month
	Inhambane	Zavala	CIS	Rural	VCT	< 170 per month

*Mavalane = Psychosocial services testing point; **Morrumbene = Triage testing point.

the health facility, agree to provide contact information, agree to adhere to study procedures and be able to provide informed consent. Study participants without access to cellular phones and those who are illiterate are included. Those without access to cellular phones enrolled at intervention sites are unable to receive the SMS component of the intervention; this information is tracked by the study staff. Exclusion criteria include being pregnant at the time of study enrollment, planning to leave the community in the next 12 months, having enrolled in HIV care in the past six months at any health facility, having initiated ART (for any duration) in the past six months at any health facility, currently taking ART, or being unable to speak or understand Portuguese or Xitsua.

Participants are recruited and enrolled immediately following HIV diagnosis at the sites. After patients receive a positive HIV test result, post-test counseling, and in the case of intervention sites, POC CD4 count testing and pre-ART counseling if eligible for ART, clinic staff informs them about the study and directs those interested in participating to study staff for more information. Study staff further explains the study and if the patient is interested, screens them for eligibility and completes the informed consent process.

Study interventions

Standard of Care (SOC)

At sites randomized to the SOC arm, patients are managed per current Mozambican Ministry of Health (MOH) guidelines (Table 2). HCT counselors refer patients diagnosed with HIV to attend HIV care services at their preferred health facility as soon as possible. In most cases, patients are verbally referred to the on-site HIV care and treatment clinic. Patients who present to the health facility receptionist to enroll in HIV care are scheduled for an appointment for clinical consultation in the on-site HIV clinic. Following the clinical consultation, patients are sent to the health facility laboratory to have blood drawn for CD4 count testing and baseline laboratory tests. Laboratory tests are processed at the health facility laboratory in four of the five

Table 2 Combination intervention strategy interventions compared to standard of care

Intervention	Standard of care	CIS (CIS, CIS + FI)	Barriers targeted by interventions
**CD4 testing**	CD4 (Cyflow, FACS Caliber/Count, POC CD4) at HIV care site laboratory if linkage completed.	POC CD4 assays at HIV testing points.	Structural
	Turnaround time: 1–4 weeks.	Turnaround time: immediate.	
**Accelerated ART initiation**	Within 1–2 months from linkage.	Accelerated ART initiation within 1 week from testing.	Biomedical
	2-3 counseling sessions, all in HIV care.	2 counseling sessions, one in HCT immediately following POC CD4 test and one in HIV care.	
	Baseline laboratory tests results obtained prior to initiation.	Draw blood for baseline laboratory tests, and initiate prior to results.	
**Cellular appointment reminders**	None.	SMS appointment reminders for all participants.	Behavioral
**Non-cash financial incentives**	None.	Pre-paid cellular air time cards.	Structural

SOC sites, and are sent off-site for processing at the remaining SOC site. Patients are asked to return for another clinical consultation approximately one to two weeks later to review laboratory results, at which point ART eligibility is determined by CD4 count and WHO stage following national guidelines: CD4 count ≤350 cells/μL and/or WHO stage III/IV. Those who are ART eligible receive on average three counseling sessions before initiating treatment. Patients receiving ART are requested to return to the health facility every two weeks for the first month, at two months, at six months, and then every six months thereafter. Patients not eligible for ART are requested to return in six months for clinical evaluation and routine laboratory testing. Peer educators follow-up with patients who miss appointments on ad hoc basis via phone or home visits.

Combination Intervention Strategy (CIS)
In order to most closely approximate "real-life" service delivery, the clinical interventions of the CIS package (i.e. POC CD4 count testing and accelerated ART initiation) are provided by clinic staff to all patients receiving care at the facility, not solely study participants. Study personnel provide SMS appointment reminders and, for participants in the CIS + FI cohort, FI only to study participants. Each of these interventions is described below.

POC CD4 count testing
Patients at the intervention sites receive a POC CD4 count test in the HCT point of service immediately following HIV diagnosis by the HCT counselor. Results are available and provided to the patient along with an explanation of their meaning within 20 minutes. All patients, regardless of CD4 count, are provided with a referral slip for the on-site HIV care clinic and instructed to enroll in care as soon as possible, although particular emphasis on prompt enrollment is provided to patients who are ART eligible as described below. Patients not yet eligible for ART who agree to enroll in HIV care immediately are escorted to the receptionist's office, whereas those who are ART eligible receive the accelerated ART initiation intervention described below.

Accelerated ART initiation for eligible patients
Patients with CD4 count ≤350 cells/μL as measured by POC CD4 count testing receive referral for accelerated ART initiation. Immediately following the POC CD4 count measurement, the HCT counselor provides the first pre-ART counseling session, in contra-distinction to the SOC arm where it is provided only after the patient links to care and receives a clinical consultation. Following the counseling session, the counselor escorts the patient to the receptionist's office for immediate enrollment in HIV care and prompt ART initiation. The receptionist expedites scheduling of ART-eligible patients for a clinical consultation so that it is scheduled as soon as possible, ideally on the same day. Patients proceed immediately to have baseline laboratory tests drawn. At the clinical consultation, the clinician evaluates the patient for ART initiation, and refers the patient for additional pre-ART counseling if necessary. Medically eligible patients are initiated on ART on the same day as their clinical consultation and prior to receipt of laboratory results.

Cellular appointment reminders
During the first month following study enrollment, study participants receive weekly reminders via text messages to link to care ("*Hi. Your health is the most important thing. Please remember to come to the health center for health services*"). For the remaining 11 months of their enrollment in the study, participants receive monthly reminder messages to remain engaged in care ("*Hi. Continue coming to the health center to take care of your and your family's health*"). In addition, participants who successfully link to HIV care receive appointment reminders tailored to their upcoming appointments ("*Hi. Your health is the most important thing. We expect to see you at your upcoming appointment scheduled for the day X*"). In order to maintain participant confidentiality, cellular appointment reminders do not refer to HIV, HIV care services, a specific health facility, or reveal any personal information. Participants are not asked to confirm receipt or reply to any messages.

Non-cash financial incentives
Participants in the CIS + FI cohort receive CIS interventions plus a series of non-cash FI in the form of pre-paid cellular air time cards. The non-cash FI air-time cards are valued at approximately $5 USD and are provided upon the following achievements: linkage into care within 1 month of testing, and engagement in care by visiting the health facility for HIV care and treatment services at six months and 12 months following study enrollment, for a total of $15 USD over the study period.

Data collection
Data collection consists of participant interviews, extraction of patient-level data from medical records, and site assessments.

Participant interviews
Participant interviews are conducted at baseline, and one month and 12 months after study enrollment by study staff using closed-ended questionnaires. The baseline questionnaire collects information on patient sociodemographics, travel to and time spent at the HIV clinic, and HIV testing history. The 1-month and 12-month questionnaires collect information on linkage to care and retention in care. All three questionnaires collect information on quality of life and social support, perceived

and experienced HIV stigma, and HIV knowledge and beliefs. Additionally, for patients in the intervention arm, the follow-up interviews assess patient receipt and acceptability of each intervention component. Baseline interviews are completed at the health facility following receipt of informed consent and contact information for follow-up interviews. For the 1-month and 12-month follow-up interviews, study staff makes up to four attempts to reach the participant by phone to complete the interview. If the participant cannot be reached by phone, study staff conducts home visits in an attempt to locate the participant and conduct the interviews. Additionally, participants who are reached by phone and indicate a preference to return to the health facility to complete the interview may do so.

Extraction of patient-level data from medical records

Upon study completion, clinical and immunological data required to construct the primary study outcomes of linkage and retention and other variables of interest are extracted from existing electronic patient-level databases. Data are also used to assess comparability of the study population to the entire HIV care clinic population enrolled during the study period.

Site assessments

Data on the configuration of HIV services at the ten study sites are collected at the beginning and at the end of the study using a standardized site assessment form.

Cost data

Costs of delivering the SOC, CIS and CIS + FI are estimated by measuring utilization rates and multiplying utilization by the resource expenditure per unit utilization: laboratories, outpatient visits, inpatient visits, patient time and transportation costs, staff time, expenses for staff training, durable equipment and non-durable equipment used by staff, and rent.

Sample size and power analysis

Based on a review of the relevant literature, under the current SOC in Mozambique we conservatively estimated that the combined outcome of linkage within 1 month and retention at 12 months after HIV diagnosis would be 25% (assuming 50% of participants enroll into HIV care within 1 month of diagnosis and 50% of these are retained in care 12 months after diagnosis). We then estimated the minimum difference we could detect in our combined outcome between CIS and SOC arms with 80% power using the Power and Sample Size (Pass 8.0) software for two independent proportions in a cluster randomization study design and a two-sided Farrington & Manning Likelihood Score Test [34], and assuming five study clusters (two sites per cluster) and 300 patients per cluster. With these assumptions, we will have 80% power to detect as statistically significant a relative risk of the combined outcome of linkage and retention between CIS and SOC arms of 1.6 (or an absolute increase in the combined outcome of 20%) comparing the CIS to SOC arms.

To address the objective of comparing the effectiveness of CIS + FI to CIS on the combined outcome of linkage within 1 month of diagnosis and 12 month retention after diagnosis, we are enrolling an additional 150 participants in each of the five CIS sites (total of 750 additional participants in intervention arm) in cohort 3 (CIS + FI) upon completion of enrollment of cohort 2 (CIS). Assuming that the combined outcome of linkage within one month of diagnosis and retention 12 months after diagnosis is 45% in the CIS arm, we will also have greater than 80% power to detect an absolute increase of 21% in the combined outcome comparing CIS + FI to CIS.

Statistical methods

1. To evaluate the effectiveness of CIS as compared to SOC on the combined outcome of linkage to HIV care within 1 month and retention in care at 12 months among adults testing HIV positive. An intent-to-treat analysis will be used to compare the combined outcome of linkage to HIV care within 1 month and retention at 12 months between participants at sites randomized to receive the SOC and participants at sites randomized to receive CIS. We will use multilevel generalized linear regression models to provide statistical estimates controlling for cluster and matched stratum effects. Models will include fixed effects for study condition (treatment arm) and random effects for sites. This approach will take into account the correlation among participants within facilities. In addition, we will compare patient-level and facility-level characteristics hypothesized to influence linkage and retention after data collection has been completed in order to assess whether the randomization evenly distributed important non-matched predictors of linkage and retention between intervention and control arms. Covariates hypothesized to influence linkage and retention that will be analyzed include: actual numbers of adults enrolling in HIV care at each site during the study period, age, gender, baseline measures of CD4 count and WHO stage at enrollment into HIV care, and tuberculosis status at enrollment into HIV care. Should important differences be observed between intervention and control arms, we will additionally conduct covariate-adjusted generalized linear mixed models to assess the degree to which these differences alter our measures of association.
2. To evaluate the incremental effectiveness of CIS + FI compared to CIS on the combined outcome of rapid

linkage to HIV care within 1 month and retention in care at 12 months among adults newly diagnosed with HIV.
The approach for this objective will follow the approach outlined under objective 1 above; in this case, the combined outcome will be compared between participants enrolling in cohort 3 (receiving CIS + FI) and those enrolling approximately six months earlier in cohort 2 (receiving CIS without FI), all within the same study arm.

3. To evaluate the effectiveness of CIS compared to SOC and the incremental effectiveness of CIS + FI compared to CIS in relation to: linkage to care within one month; retention 12 months after HIV diagnosis; time from eligibility to ART-initiation, and disease progression. Outcomes will be compared under an "intent to treat" scenario and, if significant differences in other determinants of these outcomes are observed between study cohorts at baseline, generalized multilevel mixed models will be used to compare outcomes between study cohorts. In addition, linkage rates and non-retention and mortality rates 12 months after HIV diagnosis will be compared between study arms using Kaplan-Meier survival analytic techniques using robust sandwich estimates to account for clustering. Participants who are lost to follow-up will be censored for mortality and morbidity analyses at the time of last known visit. For the subset of participants determined ART eligible at their site, Cox Proportional Hazards Models will be used to compare the time from ART eligibility to ART initiation between participants receiving CIS + FI, CIS and SOC, censoring participants transferring after ART eligibility but before initiation and treating participants who die before ART initiation as competing risks, and using robust sandwich estimates to account for clustering.
4. To evaluate the cost-effectiveness of CIS compared to SOC and the incremental cost-effectiveness of CIS + FI compared to CIS.
The incremental cost-effectiveness ratio is defined as the incremental change in costs divided by the incremental change in effects. This study will use cost-effectiveness to compare SOC with CIS, CIS with CIS + FI, and SOC with CIS + FI. The incremental cost-effectiveness of CIS compared to SOC is equal to the cost of CIS minus the cost of SOC divided by the effectiveness of CIS minus the effectiveness of SOC. In discounting future benefits expected from long-term benefits of the proposed intervention (such as improved life expectancy), we will explore alternative time horizons (ranging from the period of data collection to five years, 10 years, 20 years, and lifetime), and we will use the standard discount rate of 3% for costs and benefits. This discount rate reflects the time preference with regard to costs and benefits (that is, a dollar today is worth more than an inflation-adjusted dollar at some time in the future). The downstream impact of CIS and CIS + FI on future costs and benefits (e.g. the potential impact of prolonging lives) will be included as additional costs and benefits.
5. To assess feasibility and participant acceptability of the interventions included in CIS and CIS + FI in enhancing linkage and retention.
To assess feasibility of CIS and CIS + FI, uptake of interventions will be assessed by calculating the proportion of participants reporting that they received each intervention during the 1- and 12-month follow-up interviews and documented within study records as having received them. To assess acceptability, participants are asked to identify whether each intervention was helpful in terms of assisting in timely linkage to care and retention after enrollment into care, the "most useful" and "least useful" intervention, and to identify what aspects of each intervention were useful and not useful. Stratified analyses will examine associations between participant socio-demographic and clinical characteristics and acceptability.

Ethics
The study protocol, including recruitment, consent, and intervention materials, was approved by the Institutional Review Board (IRB) at Columbia University (FWA00002636) and by Mozambique's National Committee for Bioethics in Health (FWA00003139) and complies with United States Agency for International Development (USAID) policy on human subjects research. Study staff has been trained in good clinical practice (GCP) and standard operating procedures (SOP). Prior to being enrolled in the study, all participants are required to provide written informed consent.

Trial status
The study was launched in April 2013 at two sites in Maputo and all sites were actively enrolling participants for the SOC arm and CIS cohort of the intervention arm by December 2013. The first round of the site assessment was conducted in April 2014. As of July 2014, enrollment is complete at eight sites (four SOC sites and four intervention sites) with a total of 1351 participants enrolled. Enrollment of CIS + FI participants began at two intervention sites in June 2014.

Discussion
Study findings have the potential to substantially transform clinic-based approaches to linkage to and retention in HIV care and ultimately impact patient health outcomes and onward HIV transmission in Mozambique

and elsewhere in SSA. Specifically, each intervention component in the CIS has proven efficacy in HIV care and treatment settings but information is lacking on an easy to deliver package of services that can improve overall performance of the HIV care continuum. We anticipate that findings from our study can help to bridge this "know-do" gap and provide evidence of the effectiveness of a combination intervention strategy, and its cost-effectiveness, in a "real-life" service delivery system. We further anticipate that evidence from this study can be used to inform policy decisions concerning national HIV care and treatment policy, both in Mozambique and in other resource-limited settings.

Indeed, to ensure local relevance of study findings, a study advisory group—comprised of representatives from the funding agency, the Ministry of Health, the Mozambican National Institute of Health, and Mozambican Association of People Living with HIV—was created prior to study launch to advise on refinement of the study interventions, study implementation, translation of findings into local practice, local and international dissemination and capacity building activities. To date, the study advisory group has met three times and numerous informal consultations have been held with individual members.

Despite intimate involvement of the Ministry of Health in the study advisory group, several factors intrinsic to the implementation science nature of the study have delayed recruitment of participants, including a strike of health worker personnel, frequent electricity shortages, recurrent absences of HCT counselors at the health facilities, and intermittent shortages of HIV testing kits and other supplies. Additionally, low motivation of health facility staff has created challenges for optimal delivery of the CIS interventions. Specifically, delays in data entry of patient information in the electronic patient-level database used to follow HIV care patients by health facility data clerks have limited the ability of study staff to send SMS reminder for scheduled follow-up appointments to all participants in the CIS arm that have linked to care. Similarly, at several health facilities, receptionists have been reluctant to change their usual procedures for scheduling patient appointments in order to expedite visits for ART eligible patients as per the accelerated ART protocol.

These challenges highlight the realities of providing high quality services and changing practices and behaviors in a complex, under-resourced health system that is struggling to address the HIV epidemic. Implementation science studies, such as the one described in this paper, are critical to identifying the gaps between evidence and practice and identifying strategies to close these gaps in order to maximize the public health impact of available interventions.

Other information

Registration

Trial registration: Clinicaltrials.gov, NCT01930084.

Protocol

The full trial protocol can be accessed directly on the Clinicaltrials.gov webpage with the registration number provided above.

Abbreviations

AIDS: Acquired immunodeficiency syndrome; ART: Antiretroviral therapy; CIS: Combination intervention strategy; FI: Financial incentive; GCP: Good clinical practice; HCT: HIV counseling and testing; HIV: Human immunodeficiency virus; IRB: Institutional review board; MOH: Ministry of health; PLWH: Person(s) living with HIV/AIDS; PMTCT: Prevention of mother-to-child transmission; POC: Point of care; SMS: Short message service; SOC: Standard of care; SOP: Standard operating procedures; TB: Tuberculosis; USAID: United States agency for international development; VCT: Voluntary counseling and testing; WHO: World health organization.

Competing interests

The authors declare that they have no competing interests.

Authors' contributions

BE, MLM, MRL, LA and DH conceived and designed the study. FA, LA, and MT collected and assembled the data. BE, ML, RS, and DG drafted the article. FA, MM, LA, MRL, MT, DH, AM, and IJ critically revised the article for important intellectual content. All authors read and approved the final manuscript.

Acknowledgments

We would like to recognize the hard work and valuable contributions of the study staff. We thank the health facilities for their dedication to research, and we gratefully acknowledge the study participants.
This study is funded by United States Agency for International Development (USAID), USAID Award Number: AID-OAA-A-12-00022. USAID is the donor and advisory institution for this study. They are not directly engaged with the study. USAID employees consequently have no contact with study participants and no access to individually identifiable private information. USAID is also represented on the Study Advisory Group.

Author details

[1]ICAP, Columbia University, New York, NY, USA. [2]Department of Epidemiology, Mailman School of Public Health, New York, NY, USA. [3]Center for Collaboration in Health, Maputo, Mozambique. [4]ICAP in Mozambique, Maputo, Mozambique. [5]Office of the United States Global AIDS Coordinator, Washington, DC, USA. [6]Mozambican National Institute of Health, Maputo, Mozambique.

Received: 18 September 2014 Accepted: 8 October 2014
Published online: 15 October 2014

References

1. Fox MP, Rosen S: **Patient retention in antiretroviral therapy programs up to three years on treatment in sub-Saharan Africa, 2007–2009: systematic review.** *Trop Med Int Health* 2010, **15**(Suppl 1):1–15.
2. Mugglin C, Estill J, Wandeler G, Bender N, Gsponer T, Egger M, Keiser O: **Meta-analysis of linkage to care from HIV diagnosis to start of ART: sub-Saharan Africa.** In *19th Conference on Retroviruses and Opportunistic Infections (CROI); 5–8 March 2012; Seattle*. 2012. Abstract 1143.
3. Rosen S, Fox MP: **Retention in HIV care between testing and treatment in ub-Saharan Africa: a systematic review.** *PLoS Med* 2011, **8**(7):e1001056.
4. Rosen S, Fox MP, Gill CJ: **Patient retention in antiretroviral therapy programs in sub-Saharan Africa: a systematic review.** *PLoS Med* 2007, **4**(10):e298.
5. McNairy ML, El-Sadr WM: **The HIV care continuum: no partial credit given.** *AIDS* 2012, **26**(14):1735–1738.
6. Mugglin C, Estill J, Wandeler G, Bender N, Egger M, Gsponer T, Keiser O, Ie DEA Southern Africa: **Loss to programme between HIV diagnosis and**

initiation of antiretroviral therapy in sub-Saharan Africa: systematic review and meta-analysis. *Trop Med Int Health* 2012, **17**(12):1509–1520.
7. Barker PM, Mphatswe W, Rollins N: **Antiretroviral drugs in the cupboard are not enough: the impact of health systems' performance on mother-to-child transmission of HIV.** *J Acquir Immune Defic Syndr* 2011, **56**(2):e45–e48.
8. Fisher JD, Smith L: **Secondary prevention of HIV infection: the current state of prevention for positives.** *Curr Opin HIV AIDS* 2009, **4**(4):279–287.
9. Fisher JD, Smith LR, Lenz EM: **Secondary prevention of HIV in the United States: past, current, and future perspectives.** *J Acquir Immune Defic Syndr* 2010, **55**(Suppl 2):S106–S115.
10. Lawn SD, Campbell L, Kaplan R, Boulle A, Cornell M, Kerschberger B, Morrow C, Little F, Egger M, Wood R: **Time to initiation of antiretroviral therapy among patients with HIV-associated tuberculosis in Cape Town, South Africa.** *J Acquir Immune Defic Syndr* 2011, **57**(2):136–140.
11. Lawn SD, Kranzer K, Wood R: **Antiretroviral therapy for control of the HIV-associated tuberculosis epidemic in resource-limited settings.** *Clin Chest Med* 2009, **30**(4):685–699. viii.
12. Makadzange AT, Ndhlovu CE, Takarinda K, Reid M, Kurangwa M, Gona P, Hakim JG: **Early versus delayed initiation of antiretroviral therapy for concurrent HIV infection and cryptococcal meningitis in sub-Saharan Africa.** *Clin Infect Dis* 2010, **50**(11):1532–1538.
13. Middelkoop K, Bekker LG, Myer L, Johnson LF, Kloos M, Morrow C, Wood R: **Antiretroviral therapy and TB notification rates in a high HIV prevalence South African community.** *J Acquir Immune Defic Syndr* 2011, **56**(3):263–269.
14. Middelkoop K, Bekker LG, Myer L, Whitelaw A, Grant A, Kaplan G, McIntyre J, Wood R: **Antiretroviral program associated with reduction in untreated prevalent tuberculosis in a South African township.** *Am J Respir Crit Care Med* 2010, **182**(8):1080–1085.
15. Kranzer K, Govindasamy D, Ford N, Johnston V, Lawn SD: **Quantifying and addressing losses along the continuum of care for people living with HIV infection in sub-Saharan Africa: a systematic review.** *J Int AIDS Soc* 2012, **15**(2):17383.
16. Amuron B, Namara G, Birungi J, Nabiryo C, Levin J, Grosskurth H, Coutinho A, Jaffar S: **Mortality and loss-to-follow-up during the pre-treatment period in an antiretroviral therapy programme under normal health service conditions in Uganda.** *BMC Public Health* 2009, **9**:290.
17. Geng EH, Bangsberg DR, Musinguzi N, Emenyonu N, Bwana MB, Yiannoutsos CT, Glidden DV, Deeks SG, Martin JN: **Understanding reasons for and outcomes of patients lost to follow-up in antiretroviral therapy programs in Africa through a sampling-based approach.** *J Acquir Immune Defic Syndr* 2010, **53**(3):405–411.
18. Maskew M, MacPhail P, Menezes C, Rubel D: **Lost to follow up: contributing factors and challenges in South African patients on antiretroviral therapy.** *S Afr Med J* 2007, **97**(9):853–857.
19. Ochieng-Ooko V, Ochieng D, Sidle JE, Holdsworth M, Wools-Kaloustian K, Siika AM, Yiannoutsos CT, Owiti M, Kimaiyo S, Braitstein P: **Influence of gender on loss to follow-up in a large HIV treatment programme in western Kenya.** *Bull World Health Organ* 2010, **88**(9):681–688.
20. Rabkin M, Austin J, Nash D: **High Patient Retention Rates in a Multinational HIV/AIDS Treatment Program: The Columbia University Mother-to-Child-Plus Experience.** In *17th Conference on Retroviruses and Opportunistic Infections (CROI); 16–19 February 2010; San Francisco.* 2010.
21. Yu JK, Chen SC, Wang KY, Chang CS, Makombe SD, Schouten EJ, Harries AD: **True outcomes for patients on antiretroviral therapy who are "lost to follow-up" in Malawi.** *Bull World Health Organ* 2007, **85**(7):550–554.
22. Faal M, Naidoo N, Makgamatha L, Venter F, Osih R: **Effect of an immediate CD4 result during VCT on patient retention in ART.** In *XVIII International AIDS Conference; 18–23 July 2010; Vienna.* 2010. Abstract TUPE0113.
23. Jani IV, Sitoe NE, Alfai ER, Chongo PL, Quevedo JI, Rocha BM, Lehe JD, Peter TF: **Effect of point-of-care CD4 cell count tests on retention of patients and rates of antiretroviral therapy initiation in primary health clinics: an observational cohort study.** *Lancet* 2011, **378**(9802):1572–1579.
24. Larson BA, Ndibongo B, Bistline K, Xulu T, Brennan A, Long L, Maotoe T, Rosen S, Fox MP: **Point-of-care CD4 testing after HIV diagnosis to reduce losses to initiation of antiretroviral therapy: an evaluation of a pilot program at the Thembu Lethu Clinic, Johannesburg, South Africa.** In *6th International AIDS Conference on HIV Pathogenesis, Treatment and Prevention; 17–20 July 2011; Rome.* 2011. Abstract no. MOPE450.
25. Emenyonu N, Muyindike W, Habyarimana J, Pops-Eleches C, Thirumurthy H, Ragland K, Bangsberg DR: **Cash transfers to cover clinic transportation costs improve retention in care in a HIV treatment program in rural Uganda.** In *17th Conference on Retroviruses and Opportunistic Infections; 16–19 February 2010; San Francisco.* 2010. Abstract no. 831.
26. **National survey on prevalence, behavioral risks, and information about HIV and AIDS (2009 INSIDA).** [http://dhsprogram.com/pubs/pdf/SR179/SR179.pdf]
27. **Mozambique 2012 Global AIDS Response Progress Report.** [http://www.unaids.org/en/dataanalysis/knowyourresponse/countryprogressreports/2012countries/ce_MZ_Narrative_Report%5b1%5d.pdf]
28. **National ART Data, December 2012.** [http://www.misau.gov.mz/index.php/hiv-sida/cat_view/82-hivsida/89-informacao-estrategica-relatorios/90-relatorios-tarv]
29. Micek MA, Gimbel-Sherr K, Baptista AJ, Matediana E, Montoya P, Pfeiffer J, Melo A, Gimbel-Sherr S, Johnson W, Gloyd S: **Loss to follow-up of adults in public HIV care systems in central Mozambique: identifying obstacles to treatment.** *J Acquir Immune Defic Syndr* 2009, **52**(3):397–405.
30. Lahuerta M, Lima J, Elul B, Okamura M, Alvim MF, Nuwagaba-Biribonwoha H, Horowitz D, Fernandes R, Assan A, Abrams EJ, El-Sadr WM, Nash D: **Patients enrolled in HIV care in Mozambique: baseline characteristics and follow-up outcomes.** *J Acquir Immune Defic Syndr* 2011, **58**(3):e75–e86.
31. Palladino C, Briz V, Bellon JM, Bartolo I, Carvalho P, Camacho R, Munoz-Fernandez MA, Bastos R, Manuel R, Casanovas J, Taveira N: **Predictors of attrition and immunological failure in HIV-1 patients on highly active antiretroviral therapy from different healthcare settings in Mozambique.** *PLoS One* 2013, **8**(12):e82718.
32. Pati R, Lahuerta M, Elul B, Okamura M, Alvim MF, Schackman B, Bang H, Fernandes R, Assan A, Lima J, Nash D, Identifying Optimal Models of HIV Care in Mozambique Study Group: **Factors associated with loss to clinic among HIV patients not yet known to be eligible for antiretroviral therapy (ART) in Mozambique.** *J Int AIDS Soc* 2013, **16**:18490.
33. Elul B, Saito S, Hoos D, Lamb M, de Lima J, Hawken M, Ntumy R, Awa-Toure M, Melaku Z, El-Sadr WM: **6- and 12-month non-retention over time among 5,690 cohorts with 316,762 patients initiating ART: 9 countries in sub-Saharan Africa.** In *19th Conference on Retroviruses and Opportunistic Infections; 5–8 March 2012; Seattle.* 2012. Abstract no. 86.
34. Donner A, Klar N: *Design and Analysis of Cluster Randomization Trials in Health Research.* London: Arnold; 2000.

doi:10.1186/s12879-014-0549-5
Cite this article as: Elul *et al.*: A combination strategy for enhancing linkage to and retention in HIV care among adults newly diagnosed with HIV in Mozambique: study protocol for a site-randomized implementation science study. *BMC Infectious Diseases* 2014 **14**:549.

Submit your next manuscript to BioMed Central and take full advantage of:

- **Convenient online submission**
- **Thorough peer review**
- **No space constraints or color figure charges**
- **Immediate publication on acceptance**
- **Inclusion in PubMed, CAS, Scopus and Google Scholar**
- **Research which is freely available for redistribution**

Submit your manuscript at
www.biomedcentral.com/submit

BioMed Central
